# Kavosh: a new algorithm for finding network motifs

**DOI:** 10.1186/1471-2105-10-318

**Published:** 2009-10-04

**Authors:** Zahra Razaghi Moghadam Kashani, Hayedeh Ahrabian, Elahe Elahi, Abbas Nowzari-Dalini, Elnaz Saberi Ansari, Sahar Asadi, Shahin Mohammadi, Falk Schreiber, Ali Masoudi-Nejad

**Affiliations:** 1 Laboratory of Systems Biology and Bioinformatics, Institute of Biochemistry and Biophysics, University of Tehran, Tehran, Iran; 2 School of Mathematics and Computer Science, University of Tehran, Tehran, Iran; 3 Center of Excellence in Biomathematics, University of Tehran, Tehran, Iran; 4 School of Biology, University of Tehran, Tehran, Iran; 5 Institute for Computer Science, Martin-Luther-University Halle-Wittenberg, Halle, Germany; 6 Leibniz Institute of Plant Genetics and Crop Plant Research (IPK), Gatersleben, Germany

## Abstract

**Background:**

Complex networks are studied across many fields of science and are particularly important to understand biological processes. Motifs in networks are small connected sub-graphs that occur significantly in higher frequencies than in random networks. They have recently gathered much attention as a useful concept to uncover structural design principles of complex networks. Existing algorithms for finding network motifs are extremely costly in CPU time and memory consumption and have practically restrictions on the size of motifs.

**Results:**

We present a new algorithm (Kavosh), for finding k-size network motifs with less memory and CPU time in comparison to other existing algorithms. Our algorithm is based on counting all k-size sub-graphs of a given graph (directed or undirected). We evaluated our algorithm on biological networks of *E. coli *and *S. cereviciae*, and also on non-biological networks: a social and an electronic network.

**Conclusion:**

The efficiency of our algorithm is demonstrated by comparing the obtained results with three well-known motif finding tools. For comparison, the CPU time, memory usage and the similarities of obtained motifs are considered. Besides, Kavosh can be employed for finding motifs of size greater than eight, while most of the other algorithms have restriction on motifs with size greater than eight. The Kavosh source code and help files are freely available at: .

## Background

Large networks, such as social networks, computer and biological networks, consisting of thousands to millions of vertices, have recently attracted much attention [1]. Biological networks, including protein-protein interaction networks, gene regulatory networks, and metabolic networks, are among those most widely studied [2-4]. In order to extract meaningful information from the vast amount of data encrypted in the networks, powerful methods for computational analysis need to be developed. Milo *et al*.(2002) proposed that the existence of specific sub-graphs that repeat themselves in a specific network or even among various networks would be consistent with the tenets of evolutionary theory. Each of these sub-graphs, defined by a particular pattern of interactions between vertices, may reflect a framework in which particular functions are achieved efficiently. These sub-graphs are called network motifs. Motifs are of notable importance largely because they may reflect functional properties. Nevertheless, as possible associated functions may be unknown initially, defining motifs independent of function and based on frequency of occurrence is commonly accepted. As such, motifs can be considered as sub-graphs, which occur at significantly higher frequencies in the network under investigation than in random networks. The task of discovering motifs in networks is known as motif-finding problem. The various proposed protocols for finding motifs are designed to identify either all possible sub-graphs or the most frequent ones.

Mfinder, Pajek, MAVisto, and FANMOD are the notable existing tools for the motif-finding problem [5-8]. Relevant features for evaluation of these tools include whether or not they can present results of analysis visually; they are capable of enumerating sub-graphs; a sampling protocol is used instead of analysis of the entire network; sub-graphs are discovered or only queried graphs are found; as well as the memory usage and time needed in each algorithm and the growth of CPU time with sub-graph size. The memory usage and CPU time determine the maximum size of sub-graphs that can be analyzed. Mfinder, the first motif-mining tool, implements two kinds of motif finding algorithms: a full enumeration and a sampling method. The sampling protocol is the faster one, that assigns probability values to motifs identified, and infers frequencies from these values [5]. It is also the only tool without the option of a visual presentation and results are only provided in the format of a text. Concerning motif discovery Pajek only offers limited functionality, because it only finds specific motifs such as triads and particular tetrads in a network [6]. FANMOD algorithm is clearly the best among these with regard to computational time [8]. For example, for enumeration of all 5-size sub-graphs in the transcriptional network of *Escherichia coli *using a laptop with a 1.5 GHz Pentium M processor and 512 MB RAM, Mfinder, MAVisto, and FANMOD requires 180, 620, and 10 seconds, respectively [8]. There are 1.4 × 10^6 ^5-size sub-graphs in this network. The only problem with FANMOD is that it can handle sub-graphs consisting maximally of eight vertices. Its memory usage increases notably both with increase in sub-graph size and network size. In addition to the mentioned tools, NeMoFinder given by J. Chen and et al. [9] is an efficient network motif finding algorithm for motifs up to size 12 only for protein-protein interaction networks, which are presented by undirected graphs. Also in the case of protein interaction networks, some clustering tools are used to simplify the motif finding problem. MCODE [10] and MULIC [11] are two clustering approaches to be used. "*Power graph analysis*" is an approach for understanding protein interaction networks features [12]. Obviously the algorithms designed for both directed and undirected graphs are more time-consuming and general. We aim to derive an algorithm with lower CPU time and less memory usage that would be capable of supporting sub-graphs of all sizes. This is particularly important for analysis of biological networks where the total number of sub-graphs growths exponentially by the size of sub-graph. Our algorithm is based on counting all sub-graphs of a given graph(both directed and undirected). For enumeration of sub-graphs in the network, a novel and efficient method is presented. We evaluate our algorithm on the biological networks: the metabolic pathway of bacteria *E. coli *[13] and the transcription network of yeast *S. cerevisiae *[14], and also non-biological networks: a real social network and an electronic network. The obtained results of our algorithm are compared with three well-known motif finding tools: Mfinder, MAVisto, and FANMOD [5,7,8]. By this comparison, we show the efficiency of our algorithm. Also, our tool can be employed for finding motifs of size greater than eight, while most of the other algorithms have restriction on the size of motifs.

## Methods

### Definitions

A network considered as a large graph consists of vertices and edges. A directed graph (or network) is usually shown by *G *= (*V, E*) where *V *is a finite set of vertices and *E *is a finite set of edges, where *E *⊆ (*V *× *V*). An edge *e *= (*u*, *v*) ∈ *E *goes from vertex *u*, the source, to another vertex *v*, the target. The vertices *u *and *v *are *incident *with the edge *e *and *adjacent *to each other. A sub-graph of the graph *G *= (*V*, *E*) is a graph *G*_*s *_= (*V*_*s*_, *E*_*s*_) where *V*_*s *_⊆ *V *and *E*_*s *_⊆ (*V*_*s *_× *V*_*s*_) ∩ *E*.

The *in-degree *and *out-degree *of a vertex is defined as the number of edges coming into the vertex and the number of edges going out of it, respectively. The *degree *of a vertex is the total number of edges it is incident to. We define the *sub-graph size *as the number of vertices present in the sub-graph.

Two sub-graphs *G*_1 _= (*V*_1_, *E*_1_) and *G*_2 _= (*V*_2_, *E*_2_) are *isomorphic *if there is a one-to-one correspondence between their vertices, and there is an edge directed from one vertex to another vertex of one sub-graph if and only if there is an edge with the same direction between the corresponding vertices in the other sub-graph.

For a particular sub-graph *G*_*P*_, all sub-graphs isomorphic to *G*_*P *_in the network are considered as *matches *of *G*_*P *_. The *frequency *of a particular directed sub-graph in an input network is the number of its *matches *in the network. In this paper, it is assumed that different *matches *can have overlap in vertices or edges. *Motifs *are defined as sub-graphs, which have higher *frequencies *in the network than in random networks of equal size.

### Algorithm

Our algorithm for finding network motifs is called Kavosh and consists of four subtasks: *Enumeration*: finding all sub-graphs of a given size that occur in the input graph; *Classification*: classifying each found sub-graph into isomorphic groups; *Random graph generation*: generating random graphs with respect to the input network (enumeration and classification are also performed on random graphs) and *Motif identification*: distinguishing motifs among all found sub-graphs on basis of statistical parameters. In Kavosh, one of the most significant subtasks is the *enumeration *part. This subtask makes Kavosh different from other algorithms. Building an implicit tree according to the restrictions that will be discussed later causes improvement in both time and memory usage. The tree structure with its restrictions ensures that each individual sub-graph is enumerated only once that leads us to an efficient solution. Also using some powerful tools such as "revolving door ordering" algorithm [15] in this subtask, is an advantage of our algorithm.

*Classification *is another major subtasks of motif finding algorithms. In Kavosh, NAUTY algorithm which is the best known tool for this subtask is used. This is another feature for the efficiency of Kavosh. The details of the subtasks are presented below:

### Enumeration

Here we present an efficient method for enumeration of sub-graphs of size *k*. For counting all *k*-size sub-graphs of a given graph *G *= (*V*, *E*) whose vertices are numerically labeled, all sub-graphs that include a particular vertex are discovered. Subsequently, this vertex is removed from the network, and the process is repeated consecutively for successive vertices.

For counting the sub-graphs of size *k *that include a particular vertex, trees with maximum depth of *k*, rooted at this vertex and based on neighborhood relationship are implicitly built. Children of each vertex include both incoming and outgoing adjacent vertices. To descend the tree, a child is chosen at each level with the restriction that a particular child can be included only if it has not been included at any upper level. After having descended to the lowest level possible, the tree is again ascended and the process is repeated with the stipulation that vertices visited in earlier paths of descendent are now considered unvisited vertices. A final restriction in building trees is that all children in a particular tree must have numerical labels larger than the label of the root of the tree.

The protocol for extracting sub-graphs can now be described in greater details. The protocol makes use of the composition operation of an integer. For extraction of sub-graphs of size *k*, all possible compositions of the integer *k *- 1 must be considered. The compositions of *k *- 1 consist of all possible manners of expressing *k *- 1 as a sum of positive integers. Summations in which the order of the summands differs are considered distinct. A composition can be expressed as *k*_2_, *k*_3 _, ... *k*_*m *_where *k*_2 _+ *k*_3 _+ ... + *k*_*m *_= *k *- 1. To count sub-graphs based on the composition, *k*_*i *_vertices are selected from the *i *-th level of the tree to be vertices of the sub-graphs (*i *= 2, 3, ... *m*). The *k *- 1 selected vertices along with the vertex at the root define a sub-graph within the network.

As an example, we can consider finding sub-graphs of size 4 (*k *= 4). All compositions of *k *- 1 = 3 need to be considered; these are (1,1,1), (1,2), (2,1) and (3). For example, sub-graphs defined by (1,1,1) would include the root vertex and one valid child vertex at each of three subsequent levels.

It is possible that for a particular level *i*, *k*_*i *_*< n*_*i*_, where *n*_*i *_is the number of vertices present at level *i*. At level *i*, *C*(*n*_*i*_, *k*_*i*_) (*C*(*n, k*) is the number of different combinations of *k *elements through *n *elements) different selection of vertices need to be considered. Here, by using the "revolving door ordering" algorithm [15] all combinations containing *k*_*i *_vertices from the *n*_*i *_vertices are selected. The "revolving door ordering" algorithm is considered the fastest algorithm for generating combinations of vertices. The pseudocode for our algorithm for the enumeration subtask, which produces all *k*-size sub-graphs present in an input graph *G *= (*V, E*), is presented in Algorithm 1 (see appendix 1).

In this algorithm, the vertex *u *defines the root of a tree. Each vertex is marked as visited, if and only if it has been observed as an adjacent of any selected vertex in the upper levels. *S*_*i *_(*i *= 0,..., *m*, *m *≤ *k*- 1) is the set of all vertices from the *i*-th level included in a particular sub-graph. The subtask *Enumerate_Vertex *is described in Algorithm 2 (see appendix 2). This algorithm enumerates all sub-graphs in which a particular vertex acts as root. In Algorithm 2, the *Validate *function (see appendix 3) used to create list of valid vertices from which vertex selection can be made is described in Algorithm 3. The *Initial_Comb *and *Next_Comb *functions make use of the "revolving door ordering" algorithm as described earlier to make vertex combination selections at each level.

The above algorithms clearly identify all *k*-size sub-graphs in the network. Also, the constrictions placed on the manner in which trees are constructed also ensure that no single sub-graph will be counted more than once. Because, if a selected vertex (vertex *v*) for the current level (level *i*) were allowed to be among vertices adjacent to vertices at levels before *i *- 1, sub-graphs would be duplicated and enumerated more than once. This is because vertex *v *could be one of the vertices selected for two different compositions of a graph of size *k*. This possibility is precluded by algorithm 3 because vertices adjacent to vertices at levels <*i *- 1, are not allowed to be candidate vertices for level *i*.

**Figure 1:**
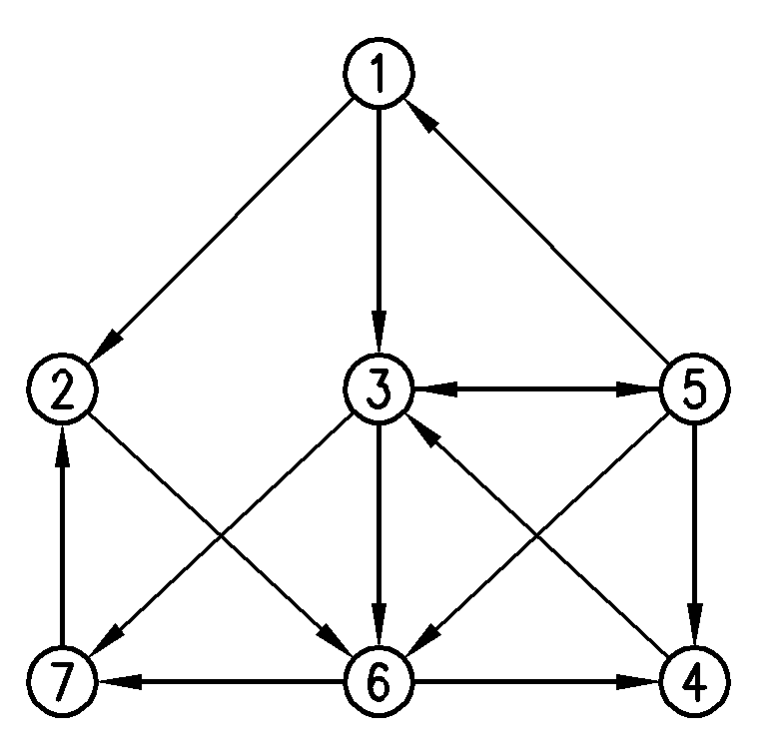
**A sample input network**. An instance of a network.

**Figure 2:**
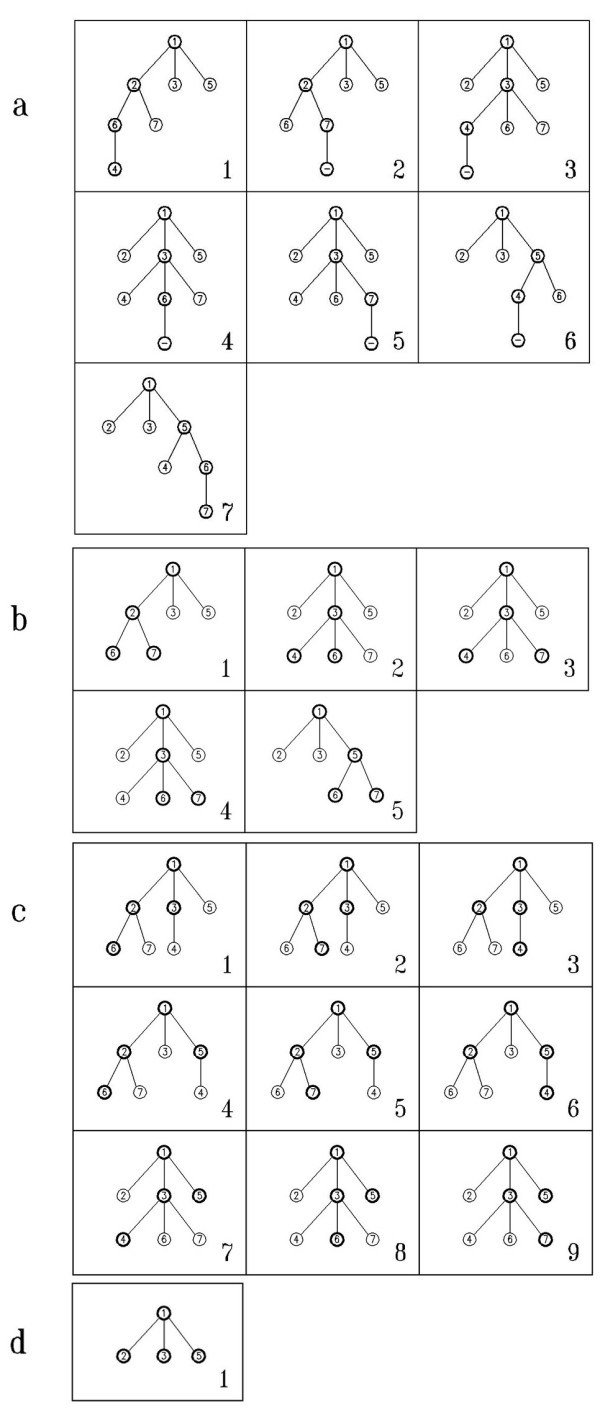
**Illustration of Kavosh algorithm**. The implicit built trees rooted at vertex 1 of size 4 for network in Figure 1.(a) Trees built according to (1,1,1) pattern. According to this pattern, after selecting vertex 1 in root, one of its neighbors must be selected, so the second selected vertex is vertex 2. Continuing the selecting process, one of the neighbors of the vertex 2 (vertex 6) and after that vertex 4 is selected. All chosen vertices are shown by specified circles in this figures. (b) Trees built according to (1,2) pattern. (c) Trees built according to (2,1) pattern. (d) Tree built according to (3) pattern.

This step is described by an example on a given graph shown in Figure 1. For this graph, all 4-size sub-graphs containing the vertex 1, are going to be found. This is illustrated in Figure 2. The vertex 1 is considered as the root of the tree and its label is considered as *visited*. As mentioned before, all the compositions of *k *- 1 = 3 are considered as the different patterns of selection. Starting with the composition (1, 1, 1) as the selecting pattern, valid children of the root are found. Due to its neighbors, the vertices 2, 3 and 5 are the valid ones, which according to the pattern one of them have to be chosen. The labels of these three vertices are now *visited*. Using the "revolving door ordering", the vertex 2 is the first chosen vertex. By using this pattern, one of the valid vertices of the vertex 2 has to be selected. The vertex 2, has three neighbors, the vertices 1,6 and 7. But the vertex 1 is previously *visited*, so it is not a valid child. So this process continues with the vertices 6 and 7, which are *visited *now. Again using "revolving door ordering", the vertex 6 is selected to be continued. As the pattern shows, one of the valid children of the vertex 6 have to be chosen as the last vertex of the sub-graph. The vertex 6 has five neighbors, the vertices 2, 3, 4, 5 and 7, but just the vertex 4 has not been visited yet, so its only valid child is the vertex 4. The vertex 4 is selected as the last vertex of the sub-graph. Now the vertices 1, 2, 6 and 4 make a sub-graph involved in the network of size 4, containing the vertex 1.

By recursively ascending the tree, for processing the other choices of selection, the lower vertices, are not *visited *anymore. So at this point, recursively ascending vertex 7, causes that the vertex 4 is not *visited *anymore. By continuing using this pattern, only one other sub-graph with vertices 1, 5, 6 and 7 is found; the details are shown in Figure 2a.

The composition (1, 2) is the next selecting pattern to be considered. The same as the previous selecting pattern, the vertices 2, 3 and 5 are the valid vertices in the first level which one of them have to be chosen according to the first element of the composition. Using "*revolving door ordering*", the vertex 2 is selected and is processed. The same as the previous pattern, the vertices 6 and 7 are the valid children of the vertex 2. Here, in this step, two vertices of this level have to be chosen according to the second element of the composition which is 2. So both the vertex 6 and 7 are selected now, and produce the sub-graph containing the vertices 1, 2, 6 and 7. Recursively ascending to level two, the next selection is the vertex 3. By ascending, the vertices 6 and 7 that became *visited *in the last step are reset to unvisited. Among all the neighbors of the vertex 3, the vertices 4, 6 and 7 are valid. Using "*revolving door ordering*", all different selections of two vertices from these three vertices are computed, which results in three different sub-graphs containing the vertices { 1, 3, 4, 6},{ 1, 3,4, 7} and { 1, 3, 6, 7}. Details are shown in Figure 2b.

In the same manner, the selecting pattern (2, 1) finds the sub-graphs containing the vertices {1, 2, 3, 6}, {1, 2, 3, 7}, {1, 2, 3, 4}, { 1, 2, 5, 6}, { 1,2, 5, 7}, { 1, 2, 5, 4}, { 1, 3, 5, 4}, { 1, 3, 5, 6} and { 1, 3, 5, 7} which is shown in Figure 2c. And using the pattern (3), the sub-graph with vertices { 1, 2, 3, 5} is found, its tree is shown in Figure 2d.

It should be noted that the reason for the efficiency of our enumeration algorithm would be the implicit tree constructed by the underlying recursion in our algorithm. The depth of this implicit recursion tree depends on the number of elements in a composition of *k*.

### Classification

**Figure 3:**
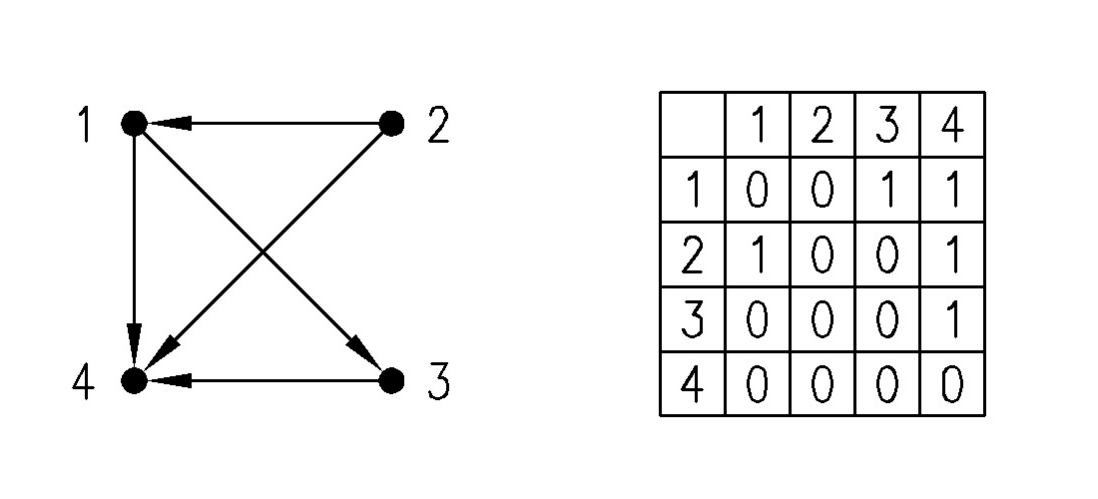
**Sample graph with its adjacency matrix**. A sample graph is shown in (a). As there are 4 vertices in this graph, there are 4! permutations on its vertices to indicate its different adjacency matrices and so different strings according to NAUTY description. The adjacency matrix in (b) reflects (1, 2, 3, 4) ordering of vertices. Among all different permutations (2, 1, 3, 4) ordering, creates the largest string which its related adjacency matrix is shown in (c) and this is the one known as *canonical labeling*.

After discovering a sub-graph involved as a match in the input network, in order to be able to evaluate the size of each class according to the input network, there is a need to classify it into isomorphic classes. The most powerful algorithm, which is usually used for finding isomorphism is NAUTY [16]. In this algorithm, a unique identifier is assigned to each class of isomorphism and called the *canonical labeling*. The *canonical labeling *is generated by the transformation of the adjacency matrix into a string by concatenating it row-by-row. As different orderings of the vertices generate different strings, an ordering of the vertices with the lexicographically largest or smallest string is chosen as *canonical labeling *between all possible permutations. As an example for the graph illustrated in Figure 3 with the corresponding adjacency matrix, the *canonical labeling *is 0101001100010000, related to the (2,1,3,4) ordering of vertices, which is the lexicographically largest string among all possible strings obtained by different orderings on vertices.

In this step of our approach, the adjacency matrix of each obtained sub-graph in the first step, is given to NAUTY as an input in order to generate its *canonical labeling *as the class identifier of that sub-graph.

This obtained identifier causes increment of the size of the corresponding class of isomorphism, by one.

### Random graph generation

According to the definition of a motif, the proper determination of sub-graph significance, needs comparison by an ensemble of appropriate random graphs. So generation of this ensemble due to a given random graph model is a necessary step of the algorithm. One of the popular random graph models on which we also focused is to preserve the degree sequence of the original graph in random graphs. There has been some researches concerning the problem of sub-graph distribution within such graphs for directed sparse random graphs [17,18]. Since biological networks are scale-free networks [4,19] the fraction of vertices having *k *edges, *p*(*k*), decays as a power law *p*(*k*) ~*k*^-*λ*^, where *λ *is often between 2 and 3, therefore they are sparse. So using this random graph model is appropriate for them.

**Figure 4:**
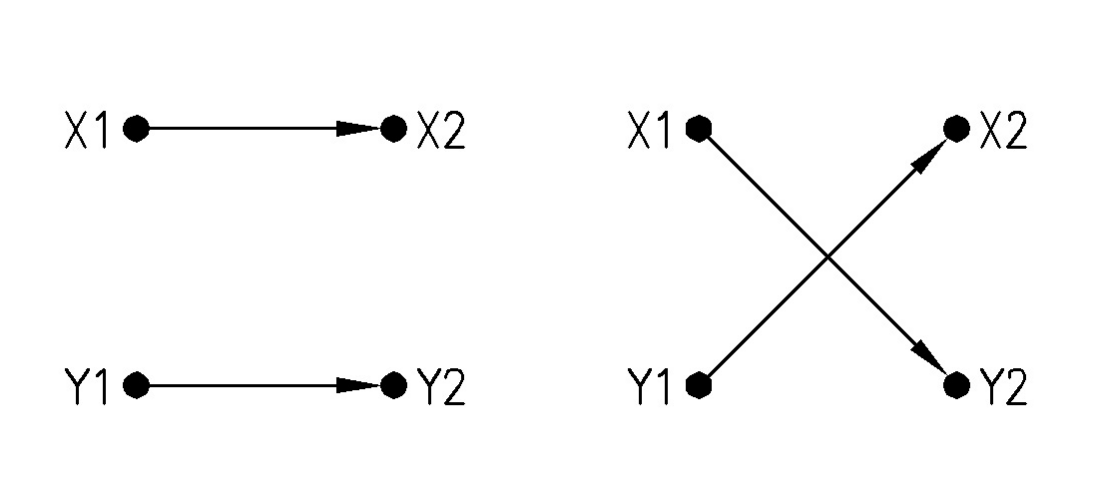
**Edge switching operator**. Edge replacement for generating random networks. As shown in this figure, the replacement process does not change the vertex degrees.

In our approach, similar to Milo's random model [17,18] switching operations are applied on the edges of the input network repeatedly, until the network is well randomized. This switching operation is applied on the randomly chosen vertices of the network as it is shown in Figure 4. By applying this switching operation repeatedly on the input network, an ensemble of random networks is generated.

For each network in the generated ensemble sub-graphs are found by using step 1 of the algorithm, and then using step 2, the size of the isomorphism classes for found sub-graphs are evaluated. This generation is necessary for comparing the real network with some random networks in order to obtain the significance of each sub-graph.

### Motif determination

By using the result of the last step, the significance of each sub-graph found in the input network is calculated. Here, some statistical measures are introduced, that lead us to the probable motifs in the input network.

### Frequency

This is the simplest measurement for estimating the significance of a motif. For a given network, assume that *G*_*P *_is a representative of an isomorphism class involved in that class. The *frequency *is defined as the number of occurrence *G*_*P *_in the input network.

### Zscore

This measure reflects how randomly the class occurred in the input network. For the assumed motif *G*_*P*_, this measure is defined as below:



where *N*_*p *_is the number which *G*_*P *_occurred in the input network,  is the mean number which *G*_*P *_occurred in random networks and *σ *is the standard deviation. The larger *Zscore*, the more significant is the motif.

### Pvalue

This measure indicates the number of random networks in which a motif *G*_*P *_occurred more often than in the input network, divided by the total number of random networks. Therefore, *Pvalue *ranges from 0 to 1. The smaller the *Pvalue*, the more significant is the motif.

These are some statistical measures implied in our algorithm to indicate the significance of a motif. For each motif found in step 1, according to the result obtained from step 2 and 3, these measures are calculated in this step.

Until now, motifs found in the input network are available including some statistical measures related to them. As mentioned in the previous step, three different measures are used in this algorithm. There are no exact thresholds for these measures to distinguish a motif, and the more restricted thresholds; the more precise is the motif. But according to the experimental results by Milo (Milo *et al*., 2002), the following conditions may be used to describe a network motif:

1. By using 1000 randomized network, the *Pvalue *is smaller than 0.01.

2. The frequency is larger than four.

3. By using 1000 randomized network, the *Zscore *is larger than one.

According to the above conditions and with respect to the sufficient preciseness, the patterns with significant measures are the ones which describe network motifs.

## Results and Discussion

In this section, we present the results of applying Kavosh to some real networks. Applications were made to network instances that are both biological and non-biological. The metabolic pathway of the bacteria *E. coli *and the transcription network of yeast *S. cereviciae *[14], a real social network, and an electronic network were targeted. These instances for testing the algorithm were up-to-date versions of the motif detection tests used by other existing algorithms (Kashtan, 2004). The biological networks, as reflected by the number of vertices therein, were notably larger than the non-biological networks used here. The numbers of sub-graphs of different sizes observed in each network are presented in Table 1. The numbers of different isomorphic groups of specific sizes observed are presented in Table 2. In all the networks, both the number of sub-graphs and the number of isomorphic groups increase exponentially with sub-graph size. Application of the FANMOD algorithm for finding sub-graphs and isomorphic groups of sizes up to eight, resulted in the identification of the same numbers as Kavosh(data not shown).

**Table 1:** Total number of sub-graphs of different sizes in different networks (rows indicate different sizes of sub-graph and columns are related to different networks).

	**3**	**4**	**5**	**6**	**7**	**8**	**9**	**10**
*E. coli*(672, 1276)	2590	12896	80724	558080	4019781	29294103	212782828	1529707241

*S. cereviciae*(688, 1079)	13150	183174	2508149	32883898	416284878	5184710063	61755820688	700928564818

*Social*(67, 182)	488	2183	10599	52156	254674	1224376	5764767	26429201

*Electronic*(97, 189)	1121	4316	19675	97038	495274	2572125	13512688	71614362

**Table 2:** Number of non-isomorphic sub-graphs in different networks (rows indicate different sizes of subgraph and columns are related to different networks).

	**3**	**4**	**5**	**6**	**7**	**8**	**9**	**10**
*E. coli*	12	83	590	3884	23587	136569	768121	4223040

*S. cereviciae*	7	34	174	888	4809	27003	183307	1083282

*Social*	13	108	773	5062	30217	165958	854023	4161577

*Electronic*	4	13	49	199	907	4333	20692	96483

**Figure 5:**
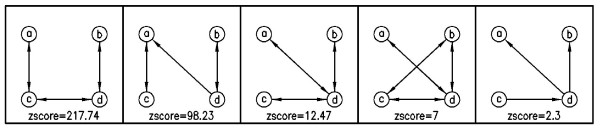
**4-size motifs of *E.Coli*, found by Kavosh**. The most significant sub-graphs of size 4 in *E. coli *network.

**Figure 6:**
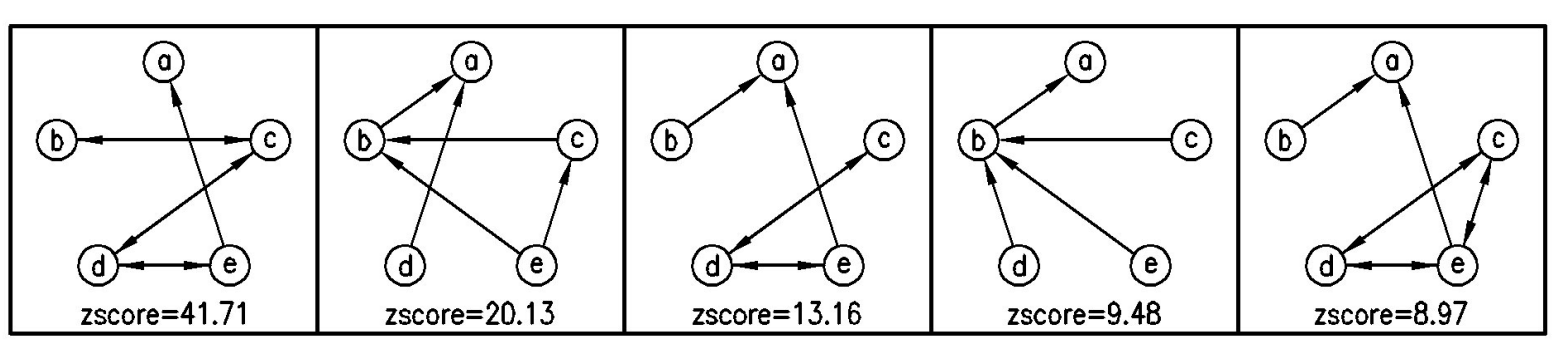
**5-size motifs of *E.Coli*, found by Kavosh**. The most significant sub-graphs of size 5 in *E. coli *network.

**Figure 7:**
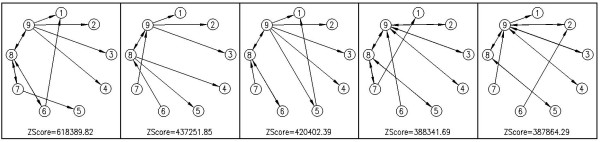
**9-size motifs of *E. Coli*, found by Kavosh**. The most significant sub-graphs of size 9 in *E. coli *network.

**Figure 8:**
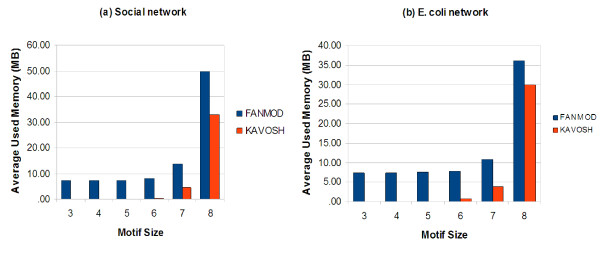
**Memory comparison**. Comparison of memory usage between FANMOD and Kavosh for two different networks (in MBytes). (a) social network. (b) *E. coli *network.

Additionally, here we present some sub-graphs, which are determined as motifs by Kavosh. We present five most significant sub-graphs of size 4, 5 and 9 in the *E. coli *network in Figures 5, 6 and 7, respectively. In this section, we aim to compare the efficiency and power of Kavosh with three previously presented programs. We apply each of the four algorithms (FANMOD, MAVisto, Mfinder and Kavosh) to the networks described. The computer system we used was equipped with a 3.2 GHz AMD Opteron processor and 8 GB RAM. For each of the real networks, 100 random networks were generated as described. Subsequently, each of the algorithms was applied to the real and all randomly generated networks. The CPU time and memory needed to perform this task was assessed for the different algorithms (Tables 3, 4, 5, and 6, and Figure 8). For all networks, the CPU time was maximum for MAVisto. As our algorithm is a full enumeration algorithm, we apply full enumeration version of Mfinder. The CPU time of Mfinder, although generally at least an order of magnitude less than that of MAVisto, was still an order of magnitude or larger than that of FANMOD and Kavosh. The CPU times of FANMOD and Kavosh were comparable for the *E. coli *network but in other networks the CPU time for Kavosh is less than the time for FANMOD (Tables 3, 4, 5, and 6). Although their time differences are sometimes not very significant, but this is because of the limitations in implementing a general motif finder tool in comparison with a limited one. Also, the time performance of Kavosh according to the number of found sub-graphs and sub-graph size in four tested network is given in table 7. This table shows the numbers of sub-graphs counted per second for each network. The largest degree is an important reason for different performances in networks. The largest degree in *S. cereviciae*, *E. coli*, electronic and social networks are respectively 71, 23, 14 and 11. As the table shows these degrees have influence in the performance. Another important aspect in this performance is that as the sub-graph size increases, the *classification *part takes more time, and this makes the algorithm slower for larger sub-graohs. In terms of memory usage, both MAVisto and Mfinder were inefficient and our computer systems could not support finding even relatively small sub-graphs, particularly in the larger tested networks. The combined effects of large CPU time and large memory usage in effect precluded size 6 sub-graph identification in even the smallest electronic network by MAVisto. Mfinder could not identify size 6 sub-graphs in the tested biological networks under the conditions of our computer system. FANMOD produced results for sub-graphs of size up to 8 in all networks used. The limitation of size 8 is inherent in the implementation protocol of FANMOD. Kavosh does not have this limitation, and the size of sub-graphs queried is only limited by computer power. Using the system described here, sub-graphs of size up to 10 were identified by Kavosh in all the networks used. For the smaller electronic network, sub-graphs of size 11 and 12 could also be identified (data not shown).

**Table 3:** Computational cost for different algorithms on the *E. coli *network (rows indicate different sizes of sub-graph and columns are related to different algorithms), times are in seconds.

	**3**	**4**	**5**	**6**	**7**	**8**	**9**	**10**
*Kavosh*	0.30	1.84	14.91	141.98	1374.01	13173.74	121110.31	1120560.16

*FANMOD*	0.81	2.53	15.71	132.24	1205.97	9256.61	-	-

*MAVisto*	13532.0	-	-	-	-	-	-	-

*Mfinder*	31.0	297.0	23671.8	-	-	-	-	-

**Table 4:** Computational cost for different algorithms on the *S. cereviciae *network (rows indicate different sizes of sub-graph and columns are related to different algorithms), times are in seconds.

	**3**	**4**	**5**	**6**	**7**	**8**	**9**	**10**
*Kavosh*	1.35	34.59	1003.92	20212.99	746385.86	17111178.28	337076691.32	7211199226.13

*FANMOD*	2.20	41.41	1111.95	24292.05	926745.34	18851135.4	-	-

*MAVisto*	15784	-	-	-	-	-	-	-

*Mfinder*	32	306	33548.2	-	-	-	-	-

**Table 5:** Computational cost for different algorithms on a social network (rows indicate different sizes of sub-graph and columns are related to different algorithms), times are in seconds.

	**3**	**4**	**5**	**6**	**7**	**8**	**9**	**10**
*Kavosh*	0.04	0.23	1.63	10.48	69.43	415.66	2594.19	14611.23

*FANMOD*	0.46	0.84	3.07	17.63	117.43	845.93	-	-

*MAVisto*	393	1492	-	-	-	-	-	-

*Mfinder*	12	49	798	181076.8	-	-	-	-

**Table 6:** Computational cost for different algorithms on an electronic network (rows indicate different sizes of sub-graph and columns are related to different algorithms), times are in seconds.

	**3**	**4**	**5**	**6**	**7**	**8**	**9**	**10**	**11**	**12**
*Kavosh*	0.08	0.36	0.02	11.39	77.22	422.614	2823.70	18037.56	135752.35	997893.27

*FANMOD*	0.53	1.06	4.34	24.24	160.00	967.99	-	-	-	-

*MAVisto*	210.0	1727.0	6696000.0	-	-	-	-	-	-	-

*Mfinder*	7.0	14.0	109.8	2020.2	-	-	-	-	-	-

**Table 7:** Performance of Kavosh on different networks(number of sub-graphs counted per second, rows indicate different sizes of sub-graph and columns are related to different networks).

	**3**	**4**	**5**	**6**	**7**	**8**	**9**	**10**
*E. coli*(672, 1276)	8633	7008	5414	3930	2925	2223	1756	1365

*S. cereviciae*(688, 1079)	9740	5295	2498	1626	557	302	183	97

*Social*(67, 182)	12200	9491	6502	4976	3668	2945	2222	1808

*Electronic*(97, 189)	14012	11988	9740	8519	6413	6093	4785	3970

The FANMOD CPU time was generally somewhat larger than that of Kavosh. Importantly, FANMOD memory usage was considerably higher than the memory usage of our Kavosh (Figure 8). In all tables, the time values are in seconds and the empty cells indicate that the algorithm cannot support that specific size or its time cannot be calculated because of the complexity.

Additionally, we present the memory usage for both Kavosh and FANMOD, which was computed with the valgrind-3.2.3 package [20]. The chart in Figure 8 compares FANMOD with Kavosh and shows that how better Kavosh works in comparison to FANMOD in this case. As it is shown in Figure 8, the growth of sub-graph numbers according to its size causes large requirement in memory. So, memory usage will be one of the problems for finding motifs of larger size.

As we can see in the tables 3, 4, 5, and 6, the only comparable algorithm with ours is FANMOD, but still is not as efficient as our algorithm. In addition to the above results, in order to show the high performance of our algorithm on large networks, we apply both Kavosh and FANMOD on Homo sapiens PPI network [21] and on Drosophila melanogaster PPI network [22], both included more than 10^4 ^nodes. Because of the high growth of the number of sub-graphs, these large networks are tested for sub-graphs of size 3, 4, and 5. The results of both Kavosh and FANMOD on Homo sapiens PPI network and Drosophila melanogaster PPI network are rfespectively shown in tables 8 and 9. As the tables show, Kavosh performs much better for larger networks.

**Table 8:** Computational cost for Kavosh and FANMOD algorithms on Homo sapiens network (times are in seconds, rows indicate different sizes of sub-graph and columns are related to different algorithms) and the numbers of sub-graphs

	**3**	**4**	**5**
*Kavosh*	15	2160	29794

*FANMOD*	36	5292	-

*Number of sub-graphs*	2750397	232652426	23287189708

**Table 9:** Computational cost for Kavosh and FANMOD algorithms on Drosophila melanogaster PPI network (times are in seconds, rows indicate different sizes of sub-graph and columns are related to different algorithms) and the numbers of sub-graphs.

	**3**	**4**	**5**
*Kavosh*	7	491	36740

*FANMOD*	10	591	39638

*Number of sub-graphs*	1201000	54112042	2963193730

## Conclusion

To improve the efficiency of our algorithm the comparison of the obtained results with three well-known motif finding tools is discussed. For comparison, the CPU time, memory usage and the similarities of obtained motifs are considered. Also, Kavosh can be employed for finding motifs of size greater than eight, while most of the other algorithms have restriction on motifs with size greater than eight. Besides, comparing with other algorithms Kavosh has better performance for large networks. In conclusion, the presented method (Kavosh) is a general motif finder that has no restrictions on motif size and also it has less time and memory consuming in comparison with other existing algorithms.

## Authors' contributions

Initial idea of the research was proposed by ZRMK, HA, AND and AMN. The Kavosh is designed, implemented, and tested by ES, SA, SM, and ZRMK. All authors participated in designing the structure and organization of the manuscript equally. All authors read and approved the final manuscript.

## Appendix: Algorithms

### Appendix 1

**Input: ***G*: input graph.

**Output: **extract all k-size sub-graphs of graph G.

1: **for each ***u *∈ *G *do

2:    *Visited *[*u*] ← **true**

3:    *S*_0 _← *u*

4:    **Enumerate_Vertex**(*G*, *u*, *S*, *k *- 1, 1)

5:    *Visited *[*u*] ← **false**

6: **end for**

Algorithm 1: Kavosh(G)

### Appendix 2

**Input: ***G*: input graph, *u*: Root vertex, *S*: selection (*S *= { *S*_0_, *S*_1_,..., *S*_*k *- 1_} is an array of the set of all *S*_*i*_), *Remainder*: number of remaining vertices to be selected,

*i*: Current depth of the tree.

**Output: **all *k*-size sub-graphs of graph *G *rooted in *u*.

1: **if ***Remainder *= 0 **then**

2:    **return**

3: **else**

4:    *V alList ← Validate(G, S*_*i*-1_, *u*)

5:    *n*_*i *_← *Min*(|*V alList*|, *Remainder*)

6:    **for ***k*_*i *_= 1 to *n*_*i *_**do**

7:       *C *← **Initial_Comb**(*V alList, k*_*i*_)

      (Make the first vertex combination selection according)

8:       **repeat**

9:          *S*_*i *_← *C*

10:          **Enumerate_Vertex**(*G, u, S, Remainder- k*_*i*_, *i *+ 1)

11:          **Next_Comb**(*V alList, k*_*i*_)

            (Make the next vertex combination selection according)

12:       **until ***C *= NILL

13:    **end for**

14:    **for each ***v *∈ *V alList ***do**

15:       *Visited *[*v*] ← **false**

16:    **end for**

17: **end if**

Algorithm 2: Enumerate_Vertex(G, u, S, Remainder, i)

### Appendix 3

**Input: ***G*: input graph, *Parents*: selected vertices of last layer, *u*: Root vertex.

**Output: **Valid vertices of the current level.

1: *V alList *← NILL

2: **for each ***v *∈ Parents **do**

3:    **for each ***w *∈ *Neighbor *[*u*] **do**

4:       **if ***label *[*u*] *< label *[*w*] AND NOT *Visited *[*w*] **then**

5:          *Visited *[*w*] ← **true**

6:          *V alList *= *V alList *+ *w*

7:       **end if**

8:    **end for**

9: **end for**

10: **return **ValList

Algorithm 3: Validate(G, Parents, u)
